# Prognostic Value of Aspartate Transaminase/Alanine Transaminase Ratio in Patients With Hepatitis B Virus-Related Hepatocellular Carcinoma Undergoing Hepatectomy

**DOI:** 10.3389/fonc.2022.876900

**Published:** 2022-05-18

**Authors:** Qiuyan Mo, Yingchun Liu, Zihan Zhou, Runwei Li, Wenfeng Gong, Bangde Xiang, Weizhong Tang, Hongping Yu

**Affiliations:** ^1^ School of Public Health, Guangxi Medical University, Nanning, China; ^2^ Research Department, Guangxi Medical University Cancer Hospital, Nanning, China; ^3^ Key Cultivated Laboratory of Cancer Molecular Medicine, Health Commission of Guangxi Zhuang Autonomous Region, Nanning, China; ^4^ Department of Cancer Prevention and Control, Guangxi Medical University Cancer Hospital, Nanning, China; ^5^ Department of Environmental and Occupational Health, School of Public Health, Indiana University, Bloomington, IN, United States; ^6^ Department of Hepatobiliary Surgery, Guangxi Medical University Cancer Hospital, Nanning, China; ^7^ Division of Colorectal & Anal Surgery, Department of Gastrointestinal Surgery, Guangxi Medical University Cancer Hospital, Nanning, China; ^8^ Guangxi Clinical Research Center for Colorectal Cancer, Nanning, China; ^9^ Key Laboratory of Early Prevention and Treatment for Regional High Frequency Tumor (Guangxi Medical University), Ministry of Education, Nanning, China; ^10^ Guangxi Key Laboratory of Early Prevention and Treatment for Regional High Frequency Tumor, Nanning, China

**Keywords:** hepatocellular carcinoma, De Ritis ratio, hepatitis B virus, prognosis, overall survival

## Abstract

**Background:**

Aspartate transaminase/alanine transaminase (De Ritis) ratio is a good predictor of liver function damage, but its prognostic value in patients with hepatocellular carcinoma (HCC) undergoing hepatectomy remains unclear. This study aimed to assess the association of the De Ritis ratio with overall survival (OS) among hepatitis B virus (HBV)-related HCC patients undergoing hepatectomy.

**Methods:**

A total of 1,147 HCC patients were recruited. Cox regression analysis was used to identify the independent risk factors. Restricted cubic spline (RCS) was used to evaluate the association between the De Ritis ratio and mortality risk. Nomogram was constructed to determine the predictive power of the De Ritis ratio.

**Results:**

Multivariate Cox regression analysis revealed that the tertile of the De Ritis ratio was an independent risk factor for mortality. After adjustment for confounding factors, the adjusted hazard ratios (HRs) with corresponding 95% CIs of mortality for the 2nd tertile and 3rd tertile were 1.175 (0.889–1.554) and 1.567 (1.199–2.046), respectively. RCS confirmed a non-linear association between the natural logarithm of the De Ritis ratio and the risk of mortality (*p* for non-linearity = 0.0375). The nomogram showed that the natural logarithm of the De Ritis ratio contributed the most to the prediction of prognosis in HBV-related HCC patients, and Harrell’s C-index was 0.680 with a 95% CI of 0.645–0.715.

**Conclusion:**

The De Ritis ratio is an independent predictor for OS in HBV-related HCC patients undergoing hepatectomy, which allows for prognostic stratification of patients, hence, individualized treatment and follow-up.

## Introduction

Liver cancer is a malignant tumor, with morbidity and mortality ranking 6th and 3rd in the world, respectively (1). Hepatocellular carcinoma (HCC) accounts for 85% to 90% of liver cancers, among which 50%–80% of cases are caused by hepatitis B virus (HBV) infection worldwide (2, 3). Hepatectomy is the recommended treatment option for patients with HCC under operable conditions and can effectively prolong the survival time of patients (4, 5). However, the overall long-term prognosis of HCC is still unsatisfactory due to high heterogeneity and high recurrence rate (6). Therefore, effective predictors to accurately assess the prognosis of HCC patients undergoing hepatectomy are an urgent need.

Aspartate transaminase (AST) and alanine transaminase (ALT) are common liver enzymes, and their elevated serum levels usually represent hepatocellular damage (7). The ratio of AST and ALT, also known as the De Ritis ratio, was first described by Fernando De Ritis in 1957 and is considered a good indicator for hepatitis etiology (8). Studies in the past decades have shown the De Ritis ratio is a good predictor of liver function damage and has been widely used to assess the causes of liver diseases such as hepatic fibrosis (9), cirrhosis (10), and non-alcoholic fatty liver disease (11). In addition, an association between the De Ritis ratio and the prognosis of HCC was reported (12). However, the results in these studies are inconsistent. Felden et al. (13) and Li et al. (14) in their studies found De Ritis ratio >2 was an independent predictor of death in HCC patients, with significantly shorter overall survival (OS) in patients with De Ritis ratio >2 than in those with De Ritis ratio ≤2. A retrospective multicenter study conducted in Taiwan showed that HCC patients with a De Ritis ratio of 1–2 and >2 had an increased risk of death as compared with those with a De Ritis ratio <1, and patients with a De Ritis ratio of >2 had the greatest risk of death (15). Lower levels of De Ritis also resulted in a better prognosis among HBV-related HCC patients when a cutoff value of De Ritis ratio = 1 was applied (16).

Currently, no universally accepted “healthy” reference interval for the De Ritis ratio has been determined. In different disease states, different De Ritis ratios may represent different etiologies (17). Thus, relationships between the De Ritis ratios in a continuous range and the prognoses of HCC need to be studied. In this study, we investigated the prognostic value of the De Ritis ratio in HBV-related HCC patients undergoing hepatectomy.

## Materials and Methods

### Patients

We retrospectively reviewed the clinical data of 2062 adult patients diagnosed with HCC at the Guangxi Medical University Cancer Hospital from February 2012 to December 2018. HBV-related HCC was defined as histopathology-confirmed HCC with positive HBV surface antigen before hepatectomy. The inclusion criteria were HCC confirmed by postoperative pathology; age from 18 to 85 years; preoperative liver function graded as Child–Pugh A or Child–Pugh B that could be improved to Child–Pugh A; complete resection of HCC; and positive hepatitis B surface antigen. The exclusion criteria included adjuvant therapies before surgery such as chemotherapy, radiotherapy, or transcatheter arterial chemoembolization (TACE); positive anti-HCV; history of other malignancies; and incomplete clinical information. This study was approved by the Ethics Committee of the Guangxi Medical University Cancer Hospital (reference no. LW2022013). Written informed consent was obtained from all patients prior to hepatectomy.

### De Ritis Ratio

ALT and AST were routinely assessed before hepatectomy in each patient, with the upper limits of normal ALT and AST levels set at 40 and 50 IU/L, respectively. The De Ritis ratio (AST/ALT) was divided into tertiles: 1st tertile ≤ 0.933, 0.933 < 2nd tertile ≤ 1.355, and 3rd tertile > 1.355.

### Endpoint and Follow-Up

The primary outcome of this study was OS. The survival status of patients was confirmed by death records or telephone calls to the patients or patients’ relatives. OS was calculated using the month unit as the duration from the surgical resection to death from any cause or the last follow-up, whichever came first. In the first 3 months postoperatively, the patients were followed up regularly at our follow-up department for monthly serum alpha-fetoprotein (AFP) levels. In the first 2 years, the patients underwent follow-up serum AFP test, chest X-ray examination, and abdominal ultrasound or contrast-enhanced CT at least every 3 months. From 3 to 5 years postoperatively, the patients were followed up every 6 months. The patients were asked to visit the outpatient clinic annually.

### Covariates

The data of patient characteristics, preoperative liver function markers, and tumor-related features were obtained from the hospital’s electronic medical record system and were included as analysis covariates. Patient characteristics included gender, ethnicity, age, body mass index (BMI), history of hypertension and diabetes mellitus (DM), Barcelona Clinic Liver Cancer (BCLC) stage, smoking status, and alcohol consumption. Status and type of hepatitis were recorded. HBV DNA was quantitatively determined and analyzed in HBV-infected patients. Preoperative serum liver enzyme tests were regularly conducted for parameters including levels of ALT, AST, total bilirubin (TBIL), and albumin, prothrombin time (PT), and the De Ritis ratio. Tumor-related clinicopathologic features included preoperative serum AFP level, tumor size, number of tumors, and tumor capsule. Patients with any missing values were excluded from subsequent processing. Finally, 1,147 patients were enrolled in the next analysis. The ALBI score formula was as follows: ALBI score = [log_10_ bilirubin (μmol/L) × 0.66] − [albumin (g/L) × 0.085]. The ALBI grades were divided into grade 1 (score ≤ −2.60), grade 2 (score >−2.60 to ≤−1.39), and grade 3 (score > −1.39) (18).

### Statistical Analysis

Categorical variables are presented as n (%) and were analyzed by the chi-square test. Continuous variables with normal distribution are reported as mean (SD) and were compared by one-way ANOVA or Student’s t-test. Non-normal distribution variables are presented as median [interquartile range (IQR)] and were analyzed by the Kruskal–Wallis H test or Mann–Whitney U test. *p*-Values for the linear trend were obtained by including the medians for each De Ritis ratio tertile as continuous variables in the linear regression models. Survival curves for tertiles of the De Ritis ratio were plotted using the Kaplan–Meier method and compared using the log-rank test. Adjusted survival curves were adjusted for covariates derived from the final model according to multivariable Cox proportional hazards models.

Univariate Cox regression analysis was used to identify potential risk factors. Multivariable Cox regression with stepwise backward selection was performed to select the independent risk factors (variables with *p* < 0.05 in univariate Cox regression subsequently entered into the model) of OS. The hazard ratio (HR) was estimated with 95% CIs, and the respective *p*-values were reported from these analyses. The De Ritis ratio was natural log-transformed due to its skewed distribution. The non-linear relationships between the natural log-transformed De Ritis ratio and OS were evaluated on a continuous scale with restricted cubic spline (RCS) curves based on Cox proportional hazards models after adjustment for confounding factors. The tests for non-linearity were calculated by Wald’s *χ* ^2^ tests. According to the Akaike information criterion, five knots were selected.

Nomogram was constructed to further investigate whether the De Ritis ratio can predict OS in patients with HBV-related HCC undergoing hepatectomy. For nomogram construction and validation, we randomly divided all the patients into training (n = 803) and validation (n = 344) cohorts in a ratio of 7:3. Univariate and multivariate Cox proportional hazards regression analyses were performed (as mentioned above) to identify variables (*p* < 0.05) that significantly affected OS in the training group. The De Ritis ratio does not have any generally accepted reference interval, and it is difficult to define the “healthy” limit (17), so we used the natural log-transformed De Ritis ratio as a predictor variable. A nomogram for predicting 3-, 5-, and 8-year survival rates in patients with HBV-related HCC undergoing hepatectomy was constructed. The nomogram was validated internally by 1,000 bootstrap resamples in the training cohort and validation in the validation cohort. Harrell’s concordance index (C-index) was calculated to evaluate the model performance for predicting outcome (19). The value of the C-index ranged from 0.5 to 1.0, with 0.5 indicating a random chance and 1.0 indicating a perfect ability to discriminate the outcome with the model. The calibration curves were used to compare the association between the actual outcomes and the predicted probabilities. Both discrimination and calibration were evaluated using bootstrapping with 1,000 resamples.

All statistical analyses were performed with STATA 15/SE statistical software (Stata Corp LP, College Station, TX, USA) and R software version 4.0.2 (R Foundation for Statistical Computing, Vienna, Austria). *p* < 0.05 (two-tailed) indicates statistical significance.

## Results

### Patient Characteristics

Baseline characteristics of 1,147 study patients stratified by tertiles of the De Ritis ratio are shown in **Table 1**. The study patients had a mean age of 49.7 (SD, 10.9 years) and a median De Ritis ratio of 1.1 (IQR, 0.9–1.5). The majority of study patients had liver cirrhosis (72.5%), were male (88.1%), and of Han ethnicity (61.3%). Male ratio, leukocyte count, hemoglobin level, albumin level, ALBI grade, AFP level, main tumor size, BCLC stage, and tumor thrombus were different among the three groups (*p* < 0.05). Overall, patients with a higher De Ritis ratio level had lower BMI and albumin levels, but a higher proportion of ALBI grade; had HBV DNA content ≥103 copies/ml, AFP level ≥400 ng/ml, and main tumor size ≥5 cm; and presented with BCLC stage B and C, and tumor thrombus (see **Table 1**).

**Table 1:** Baseline characteristics of the study patients according to tertiles of De Ritis ratio.

Characteristics	Overall (N = 1,147)	1st tertile (N = 384)	2nd tertile (N = 381)	3rd tertile (N = 382)	*p*-Value	*p* for trend^a^
Age [years, mean (SD)]	49.7 (10.9)	48.1 (10.2)	50.9 (10.9)	50.0 (11.4)	0.097	0.143
Male, n (%)	1,011 (88.1)	365 (95.1)	341 (89.5)	305 (19.8)	<0.001	<0.001
Ethnicity, n (%)					0.228	0.114
Han	703 (61.3)	248 (64.6)	231 (60.6)	224 (58.6)		
Others	444 (38.7)	136 (35.4)	150 (39.4)	158 (41.4)		
Smoking, n (%)	443 (38.6)	155 (40.4)	153 (40.2)	135 (35.3)	0.272	0.706
Alcohol consumption, n (%)	367 (32.0)	135 (35.2)	120 (31.5)	112 (29.3)	0.216	0.845
DM, n (%)	50 (4.4)	24 (6.3)	15 (3.9)	11 (2.9)	0.065	0.014
Liver cirrhosis, n (%)	831 (72.5)	286 (74.5)	279 (73.23)	266 (69.6)	0.297	0.238
BMI [kg/m^2^, mean (SD)]	22.4 (3.3)	23.2 (3.2)	22.3 (3.2)	21.7 (3.2)	0.914	<0.001
Leukocyte [10^9^/L, mean (SD)]	6.4 (2.1)	6.5 (1.9)	6.4 (2.1)	6.4 (2.3)	0.001	0.503
Hemoglobin [g/L, mean (SD)]	131.2 (50.8)	133.0 (36.6)	135.8 (70.9)	124.9 (36.8)	<0.001	0.088
Albumin [g/L, mean (SD)]	39.5 (5.1)	40.3 (5.1)	39.6 (5.2)	38.5 (4.6)	0.042	<0.001
TBIL [μmol/L, median (IQR)]	13.3 (9.8–17.6)	13.3 (9.9–17.7)	13.0 (9.4–17.0)	13.8 (10.1–18.2)	0.165	0.229
ALBI score [mean (SD)]	−2.6 (0.5)	−2.7 (0.5)	−2.6 (0.5)	−2.5 (0.4)	0.656	<0.001
ALBI grade, n (%)					0.003	
Grade 1	596 (52.0)	219 (57.0)	209 (54.9)	168 (44.0)		0.001
Grade 2	540 (47.1)	162 (42.2)	170 (44.6)	208 (54.5)		0.003
Grade 3	11 (1.0)	3 (0.8)	2 (0.5)	6 (1.6)		0.137
AST [U/L, median (IQR)]	41.0 (31.0–58.0)	39.0 (29.0–51.0)	39.0 (31.0–54.0)	46.5 (34.0–70.0)	0.0001	<0.001
ALT [U/L, median (IQR)]	37.0 (26.0–54.0)	53.0 (39.0–74.0)	35.0 (27.0–49.0)	25 (19.0–36.0)	0.0001	<0.001
HBV DNA [copies/ml, n (%)]					0.099	0.049
<10^3^	381 (33.2)	115 (30.0)	124 (32.6)	142 (37.2)		
≥10^3^	766 (66.8)	269 (70.1)	257 (67.5)	240 (62.8)		
Child–Pugh, n (%)					0.064	0.018
A	1,080 (94.2)	367 (95.6)	362 (95.0)	351 (91.9)		
B	67 (5.8)	17 (4.4)	19 (4.99)	31 (8.1)		
AFP [ng/ml, n (%)]					<0.001	<0.001
<400	648 (56.5)	246 (64.1)	222 (58.3)	180 (47.1)		
≥400	499 (43.5)	138 (35.9)	159 (41.7)	202 (52.9)		
PT [s, n (%)]					0.153	0.068
≤14	947 (82.6)	327 (85.2)	315 (82.7)	305 (79.8)		
>14	200 (17.4)	57 (14.8)	66 (17.3)	77 (20.2)		
Tumor number, n (%)					0.339	0.181
Solitary	869 (75.8)	301 (78.4)	284 (74.5)	284 (74.4)		
Multiple	278 (24.2)	83 (21.6)	97 (25.5)	98 (25.7)		
Main tumor size [cm, n (%)]					<0.001	<0.001
<5	407 (35.5)	177 (46.1)	151 (39.6)	79 (20.7)		
≥5	740 (64.5)	207 (53.9)	230 (60.4)	303 (79.3)		
BCLC stage, n (%)					<0.001	<0.001
0–A	566 (49.4)	222 (57.8)	183 (48.0)	161 (42.2)		
B–C	581 (50.7)	162 (42.2)	198 (52.0)	221 (57.9)		
Tumor capsule, n (%)	772 (67.3)	259 (67.5)	260 (68.2)	253 (66.2)	0.837	0.629
Tumor thrombus, n (%)	320 (27.9)	81 (21.1)	97 (25.5)	142 (37.2)	<0.001	<0.001
De Ritis ratio [median (IQR)]	1.1 (0.9–1.5)	0.8 (0.7–0.9)	1.1 (1.0–1.2)	1.7 (1.5–2.2)	0.0001	<0.001

OS, overall survival; IQR, interquartile range; DM, diabetes mellitus; AFP, alpha-fetoprotein; PT, prothrombin time; BMI, body mass index; TBIL, total bilirubin; ALBI, albumin–bilirubin; AST, aspartate transaminase; ALT, alanine transaminase; HBV, hepatitis B virus; BCLC, Barcelona Clinic Liver Cancer; De Ritis ratio, aspartate transaminase/alanine transaminase ratio.

a Adjusted for age (years), gender (male, female), and race (Han, others).

### Association of the De Ritis Ratio With Overall Survival

**Figure 1:**
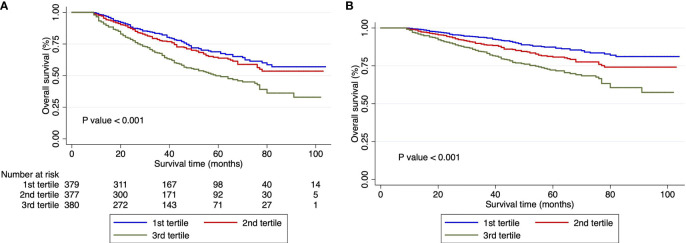
Survival curves in patients with hepatocellular carcinoma undergoing hepatectomy according to De-Ritis ratio tertiles. **(A)** Unadjusted Kaplan–Meier survival curves. **(B)** Adjusted survival Kaplan–Meier curves; curves were adjusted for Barcelona Clinic Liver Cancer stage, albumin–bilirubin grade, cancer thrombus, tumor capsule, and hepatitis B virus DNA. Ratio, tertiles of the aspartate transaminase/alanine transaminase ratio.

In this study, the median follow-up was 35 months (range, 3 days to 104 months). A total of 346 patients met the endpoint events over 8 years after surgery, with a median survival time of 80 months. Through the Kaplan–Meier survival curves, we found that the death risk increased as the level of the De Ritis ratio increased to 3rd tertiles (**Figure 1A**). The difference between the survival curves was statistically significant (*p* < 0.001). After further adjustment for confounding factors, the association of the De Ritis Ratio with OS was still statistically significant (*p* < 0.001) (**Table 2** and **Figure 1B**).

**Table 2:** Univariable and multivariable Cox regression analyses of factors associated with OS in study patients (N = 1,147).

Variables	Univariate			Multivariable		
	*β*	HR (95% CI)	*p*-Value	*β*	HR (95% CI)	*p*-Value
Age, years	−0.004	0.996 (0.986–1.005)	0.370			
Gender, male vs. female	0.357	1.430 (0.999–2.048)	0.051			
Ethnicity, others vs. Han	0.231	1.260 (1.019–1.558)	0.033			
Smoking, present vs. absent	0.303	1.354 (1.092–1.677)	0.006			
Alcohol consumption, present vs. absent	0.133	1.142 (0.911–1.432)	0.248			
DM, yes vs. no	−0.094	0.910 (0.551–1.504)	0.714			
Liver cirrhosis, present vs. absent	0.260	1.297 (1.013–1.661)	0.039			
AFP, ≥400 vs. <400 ng/ml	0.370	1.448 (1.173–1.788)	0.001			
PT, >14 vs. ≤14 s	0.172	1.188 (0.922–1.531)	0.184			
BMI, kg/m^2^	−0.026	0.974 (0.943–1.007)	0.125			
Leukocyte, 10^9^/L	0.059	1.060 (1.011–1.113)	0.017			
Hemoglobin, g/L	−0.004	0.996 (0.993–0.999)	0.013			
ALBI grade						
Grade 2 vs. grade 1	0.561	1.752 (1.408–2.179)	<0.001	0.476	1.610 (1.293–2.006)	<0.001
Grade 3 vs. grade 1	1.070	2.917 (1.077–7.896)	0.035	0.927	2.528 (0.931–6.867)	0.069
HBV DNA, ≥10^3^ vs. <10^3^ copies/ml	0.471	1.602 (1.247–2.057)	<0.001	0.366	1.442 (1.122–1.854)	0.004
Tumor number, multiple vs. solitary	0.456	1.578 (1.260–1.977)	<0.001			
Child–Pugh, B vs. A	0.510	1.665 (1.124–2.467)	0.011			
Main tumor size, ≥5 vs. <5 cm	0.620	1.859 (1.461–2.366)	<0.001			
BCLC stage, B–C vs. 0–A	0.901	2.462 (1.967–3.081)	<0.001	0.624	1.866 (1.461–2.384)	<0.001
Tumor capsule, absent vs. present	0.619	1.858 (1.501–2.300)	<0.001	0.292	1.339 (1.068–1.679)	0.011
Tumor thrombus, present vs. absent	0.809	2.244 (1.813–2.780)	<0.001	0.439	1.551 (1.225–1.964)	<0.001
De Ritis ratio						
2nd vs. 1st tertile	0.161	1.175 (0.889–1.554)	0.259	0.110	1.117 (0.843–1.479)	0.442
3rd vs. 1st tertile	0.656	1.927 (1.487–2.497)	<0.001	0.449	1.567 (1.199–2.046)	0.001

OS, overall survival; HR, hazard ratio; DM, diabetes mellitus; AFP, alpha-fetoprotein; PT, prothrombin time; BMI, body mass index; TBIL, total bilirubin; INR, international normalized ratio; ALBI, albumin–bilirubin; HBV, hepatitis B virus; BCLC, Barcelona Clinic Liver Cancer; De Ritis ratio, aspartate transaminase/alanine transaminase ratio.

Univariate and multivariate Cox regression analyses demonstrated that the tertile of the De Ritis ratio was an independent prognostic risk factor for patients with HBV-related HCC undergoing hepatectomy. Patients in the 1st tertile had no significantly different risk of death from patients in the 2nd tertile (HR = 1.175, 95% CI: 0.889–1.554), while patients in the 3rd tertile had a significantly greater risk of death (HR = 1.927, 95% CI: 1.1.487–2.497). After further adjustment for confounding factors, the HRs of mortality for the 2nd and 3rd tertiles were 1.117 (95% CI: 0.843–1.479) and 1.567 (95% CI: 1.199–2.046), respectively. The analyses also showed that ALBI grade, BCLC stage, HBV DNA content, deficient tumor capsule, and tumor thrombus were independent risk factors for the prognosis (**Table 2**).

**Figure 2:**
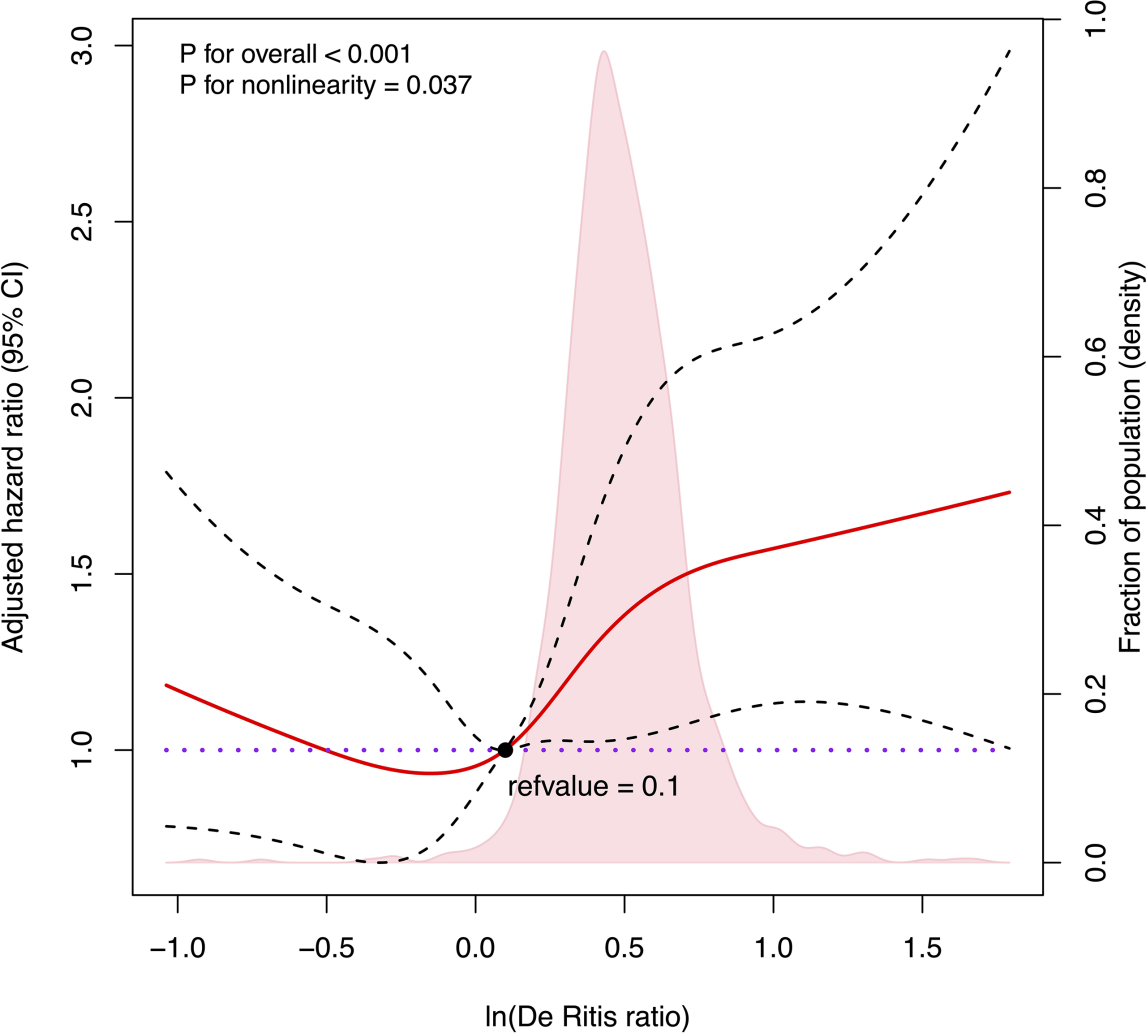
Cubic spline plot of the association between the natural logarithm of De Ritis ratio and the overall survival among patients with hepatocellular carcinoma undergoing hepatectomy. The solid red line and black dashed line represent the multivariable-adjusted hazard ratios and their corresponding 95% CIs derived from restricted cubic spline regressions with five knots. Pink shade shows fraction of population with different levels of De Ritis ratio. The black solid dot (refvalue = 0.1) indicates the natural logarithm of De Ritis ratio level with a risk of death of 1.0. Reference lines for no association are indicated by the purple dot-dashed line at a hazard ratio of 1.0. Analyses were adjusted for Barcelona Clinic Liver Cancer stage, albumin–bilirubin grade, cancer thrombus, tumor capsule, and hepatitis B virus DNA. ln(De Ritis ratio), the natural logarithm of aspartate transaminase/alanine transaminase ratio.)

We further used RCS to visualize the association between the natural logarithm of the De Ritis ratio on a continuous scale and the risk of mortality in patients with HBV-related HCC undergoing hepatectomy. In the low level of the natural logarithm of the De Ritis ratio (a De Ritis ratio <1.1), the 95% CI included the HR of 1.0 at any level of the natural logarithm of the De Ritis ratio to mortality. High levels (a De Ritis ratio ≥1.1) were associated with an increased risk of mortality, and as the ratio continued to increase, the risk of death showed a significant upward trend and then leveled off (**Figure 2**, *p* for non-linearity = 0.0375).

### Nomogram Construction and Validation

There were no significant differences in demographic and clinical characteristics between the training and validation cohorts (**Table S1**). The results of univariate and multivariate Cox regression analyses in the training cohort are shown in **Table 3**. All significant factors (*p* < 0.05) in the univariable analysis were entered into the multivariable analysis. Multivariate Cox regression analysis revealed that smoking status, ALBI grade, HBV DNA content, BCLC stage, tumor thrombus, and ln(De Ritis ratio) were selected as predictors for the nomogram (**Table 3**).

**Table 3:** Univariable and multivariable Cox regression analyses of factors associated with OS in the training set (N = 803).

Variables	Univariate			Multivariable		
	*β*	HR (95% CI)	*p*-Value	*β*	HR (95% CI)	*p*-Value
Age, years	−0.009	0.991 (0.979–1.002)	0.121			
Gender, male vs. female	0.458	1.581 (1.020–2.450)	0.040			
Ethnicity, others vs. Han	0.154	1.167 (0.907–1.500)	0.229			
Smoking status, present vs. absent	0.399	1.490 (1.158–1.916)	0.002	0.331	1.393 (1.081–1.796)	0.011
Alcohol consumption, present vs. absent	0.084	1.087 (0.833–1.420)	0.539			
DM, yes vs. no	−0.312	0.732 (0.389–1.378)	0.333			
Liver cirrhosis, present vs. absent	0.230	1.259 (0.938–1.690)	0.126			
AFP, ≥400 vs. <400 ng/ml	0.341	1.406 (1.095–1.806)	0.008			
PT, >14 vs. ≤14 s						
BMI, kg/m^2^	−0.026	0.974 (0.938–1.013)	0.187			
Leukocyte, 10^9^/L	0.081	1.084 (1.024–1.147)	0.005			
Hemoglobin, g/L	−0.003	0.997 (0.993–1.000)	0.062			
ALBI grade						
Grade 2 vs. grade 1	0.451	1.570 (1.213–2.033)	0.001	0.443	1.558 (1.202–2.020)	0.001
Grade 3 vs. grade 1	1.306	3.692 (1.165–11.692)	0.026	1.246	3.477 (1.093–11.062)	0.035
INR	0.073	1.076 (0.802–1.443)	0.627			
HBV DNA, ≥10^3^ vs. <10^3^ copies/ml	0.509	1.663 (1.232–2.246)	0.001	0.400	1.492 (1.103–2.018)	0.009
Tumor number, multiple vs. solitary	0.362	1.437 (1.098–1.879)	0.008			
Child–Pugh, B vs. A	0.622	1.862 (1.178–2.942)	0.008			
Main tumor size, ≥5 vs. <5 cm	0.649	1.913 (1.434–2.552)	<0.001			
BCLC stage, B–C vs. 0–A	0.967	2.630 (2.006–3.447)	<0.001	0.713	2.040 (1.526–2.727)	<0.001
Tumor capsule, absent vs. present	0.595	1.813 (1.409–2.332)	<0.001			
Tumor thrombus, present vs. absent	0.849	2.338 (1.818–3.007)	<0.001	0.546	1.727 (1.313–2.272)	<0.001
ln(De Ritis ratio)	0.405	1.500 (1.176–1.913)	0.001	0.248	1.282 (1.009–1.630)	0.042

OS, overall survival; HR, hazard ratio; DM, diabetes mellitus; AFP, alpha-fetoprotein; PT, prothrombin time; BMI, body mass index; TBIL, total bilirubin; INR, international normalized ratio; ALBI, albumin–bilirubin; HBV, hepatitis B virus; BCLC, Barcelona Clinic Liver Cancer; ln(De Ritis ratio), the natural logarithm of aspartate transaminase/alanine transaminase ratio.

**Figure 3:**
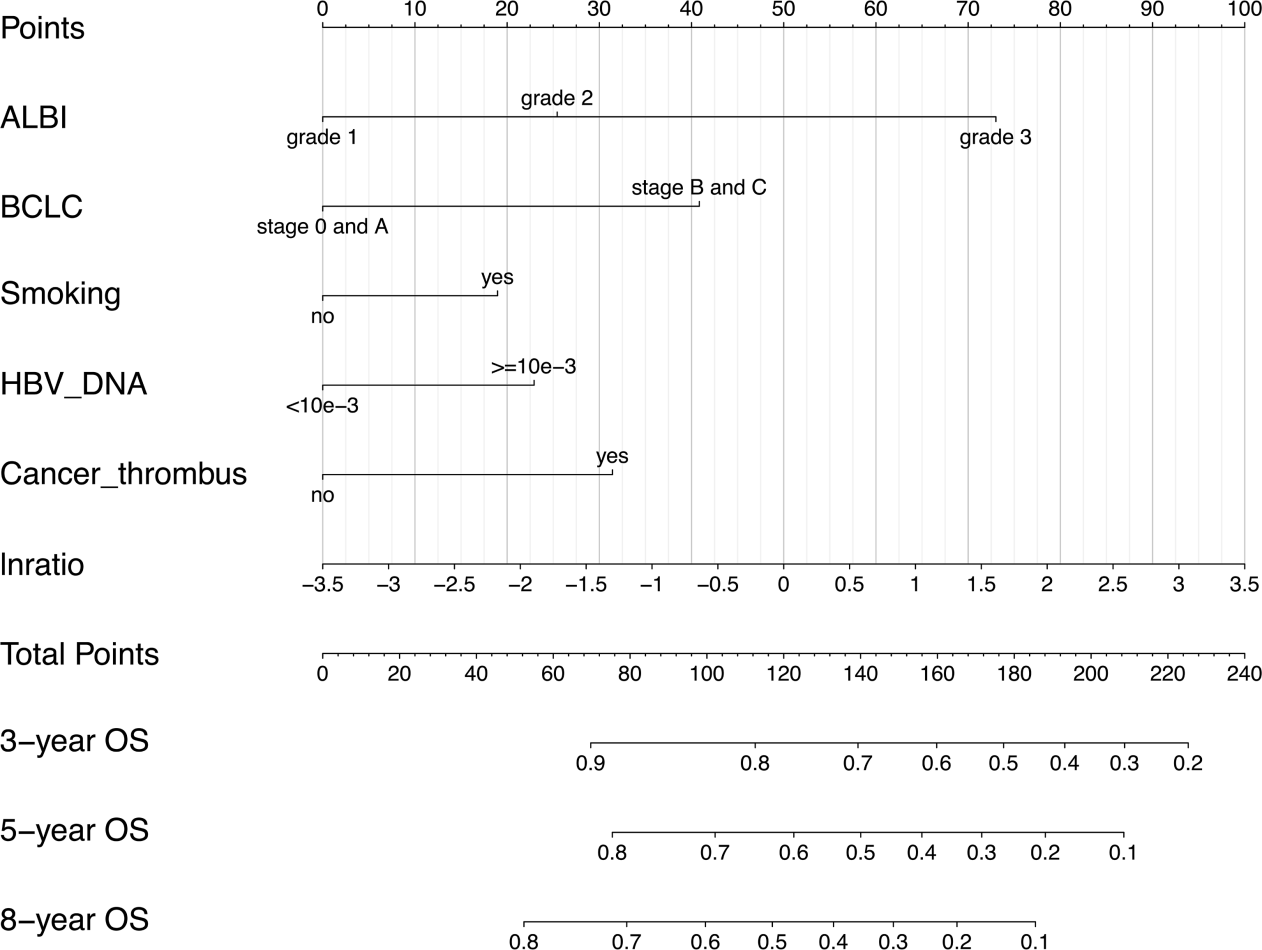
Postoperative prognostic nomogram for patients with hepatocellular carcinoma. ALBI, albumin–bilirubin; BCLC, Barcelona Clinic Liver Cancer; lnratio, the natural logarithm of aspartate transaminase/alanine transaminase ratio.

**Figure 4:**
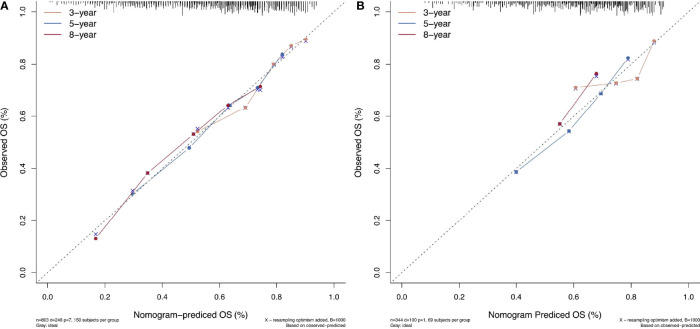
The calibration curves for predicting patient survival at each time point in the **(A)** training set and **(B)** validation set. Nomogram-predicted overall survival (OS) is plotted on the x-axis; actual OS is plotted on the y-axis. A plot along the 45° line indicates a perfect calibration model in which the predicted probabilities are identical to the actual outcomes.

All the 6 abovementioned variables were used to draw a postoperative nomogram to predict 3-, 5-, and 8-year OS (**Figure 3**). The nomogram showed that ln(De arthritis ratio) contributed the most to the prediction of prognosis in patients, followed by ALBI grade and BCLC stage. Harrell’s C-index of a prognostic nomogram for OS was 0.680 (95% CI: 0.645–0.715) in the training cohort and 0.637 (95% CI: 0.578–0.695) in the validation cohort. Overall, the calibration curves fitted well between the nomogram prediction and actual observation for 3-, 5-, and 8-year OS in the training and validation cohorts (**Figure 4**).

## Discussion

The serum levels of AST and ALT, two key enzymes in the biological process, are routinely assessed for liver function prior to clinical treatment (20). The De Ritis ratio (AST/ALT) was initially thought to be a valuable diagnostic marker for the etiology of hepatitis (e.g., acute viral hepatitis) (8), while the association between an elevated De Ritis ratio and poor prognosis of various cancers has been subsequently found (21–24), including HCC (25–27). However, studies about the association between a baseline De Ritis ratio and prognosis of HCC patients undergoing hepatectomy are limited, and the results are inconsistent. In this study, the Kaplan–Meier survival analysis showed that postoperative mortality risk increased with a preoperative De Ritis ratio in the patients with HBV-related HCC. We conclude that the De Ritis ratio is an independent prognostic risk factor for patients with HBV-related HCC undergoing hepatectomy. This finding was further supported by our results based on RCS modeling that flexibly examined the association between continuous De Ritis ratios and mortality risks.

The exact cutoff value of the De Ritis ratio to predict the prognosis of HCC patients remains undetermined despite many studies. In this study, we reported no association between a De Ritis ratio <1.1 and postoperative mortality risk, while the ratio ≥1.1 was positively related to postoperative mortality risk. However, the increase in postoperative mortality risk was not unlimited but would decrease gradually and level off once a certain level was reached, which is in line with our physiological interpretation. Similarly, in the study of Liu et al. of HCC patients undergoing TACE, the receiver operating characteristic (ROC) curve identified the cutoff value of the De Ritis ratio as 1.2. The patients with a De Ritis ratio greater than 1.2 had much lower survival rates at 1, 3, and 5 years after TACE than did those with a De Ritis ratio equal to or lower than 1.2 (28). Our results suggest that a baseline De Ritis ratio equal to or greater than 1.1 may serve as the cutoff value for possible adverse postoperative outcomes in HCC patients undergoing hepatectomy. Moreover, elevated AST level was more responsible for poor postoperative prognosis in HCC patients than was ALT.

The underlying pathophysiology mechanisms by which an elevated De Ritis ratio is associated with poor prognosis in patients with HBV-related HCC remains unclear. Altered cellular metabolism is a hallmark of cancer, and the levels of AST and ALT may be affected by cancer-associated metabolic disorders. ALT mainly exists in the cytoplasm of hepatocytes, while AST is present in both the cytoplasm and mitochondria of hepatocytes. Mitochondria play a major role in producing ATP by oxidative phosphorylation, but in the tumor microenvironment, mitochondria in tumor cells generate energy through glycolysis, thereby affecting oxidative metabolism (29). Mitochondria provide a basis for tumor anabolism, redox control, calcium homeostasis, transcriptional regulation, and cell death control (30). Furthermore, HBV X protein (HBx) induces epigenetic silencing of NAD(P)H:quinone oxidoreductase 1 (NQO1) in hepatoma cells through promoter hypermethylation. NQO1 participates in the detoxification of dopamine-derived quinones and reactive oxygen species, while downregulation of NQO1 reduces intracellular glutathione levels, thereby impairing mitochondrial function and increasing the susceptibility of hepatoma cells to oxidative stress-induced cellular damage (31). Based on these mechanisms, high tumor progression activity and impaired mitochondrial function may cause additional AST release into the bloodstream, resulting in a dramatic increase in serum AST levels. On the other hand, the clearance of AST decreases as the liver function deteriorates. Thus, an elevated De Ritis ratio at baseline reflects tumor progression and deterioration of liver functional reserve. However, the transient elevation of serum transaminases in HCC patients after superselective transarterial chemoembolization was not accompanied by deterioration of liver functional reserve compared with baseline values but was secondary to tumor necrosis resulting in the release of large amounts of serum transaminases, reflecting a response to therapy (32).

We also identified that ALBI grade, BCLC stage, HBV DNA content, deficient tumor capsule, and tumor thrombus are independent risk factors for the prognosis in patients with HBV-related HCC undergoing hepatectomy. These findings are highly consistent with previous reports on the prognostic risk factors of HCC (13, 15, 25, 28). ALBI grade, a new measure for liver function, was thought to be an ideal alternative for Child–Pugh classification due to its higher accuracy in predicting the OS in HCC patients undergoing hepatectomy (33). BCLC staging was utilized to evaluate the prognosis of HCC patients and establish subsequent treatment strategies according to prognostic subtypes (34). A high level of HBV DNA is known to be associated with progressive liver diseases. Studies demonstrated that high HBV DNA load and ALBI score could predict a poor prognosis for HCC patients (35). In previous studies, a deficient tumor capsule could predict the MVI of HBV-related HCC as a significant prognostic factor in HCC patients undergoing hepatectomy (36). In our study, patients with encapsulated HCC had a fair prognosis. That tumor thrombus as a common complication of tumors resulted in a poor prognosis of HCC is consistent with the finding in a previous study (37).

The nomogram in this study revealed that the baseline De Ritis ratio had a superior predictive value for the prognosis in patients with HBV-related HCC, which agrees with the finding in a meta-analysis that a preoperative De Ritis ratio could serve as a valuable prognostic factor for patients with malignant tumors (12). Most importantly, our results indicated that the De Ritis ratio contributed the most to the prediction of prognosis in patients with HBV-related HCC undergoing hepatectomy, as compared with other included predictors. The De Ritis ratio can be easily accessible, and its detection is affordable owing to the routine examination of AST and ALT prior to clinical treatment. Therefore, we believe the De Ritis ratio can serve as a new biomarker to accurately predict the prognosis in patients with HBV-related HCC undergoing hepatectomy. Notably, Liu et al. reported that the combination of the De Ritis ratio and the neutrophil–lymphocyte ratio (NLR) may improve the prognostic accuracy in HCC patients undergoing TACE (28). Therefore, a more extensive evaluation of the prognostic role of the De Ritis ratio in patients with HBV-related HCC is required.

As far as we know, this is the first study designed to assess the impact of a baseline De Ritis ratio on OS in patients with HBV-related HCC undergoing hepatectomy, but several limitations should be considered. First, this is a single-center retrospective study with biased results; thus, a multicenter prospective study is needed to verify the prognostic value of the De Ritis ratio in patients with HBV-related HCC undergoing hepatectomy. Second, disorders pertaining to the thyroid and muscular system as well as hemolysis may increase serum AST levels (7), although non-liver-related diseases that cause elevated AST levels can be excluded by clinical examination and biochemical tests. Third, alcohol consumption is a negative prognostic factor of mortality in HBV patients (38); although the association between alcohol consumption history and OS was not statistically significant in this study, the effect of alcohol consumption could not be completely excluded.

In conclusion, our results demonstrated that the preoperative De Ritis ratio is a good predictor of the prognosis of patients with HBV-related HCC undergoing hepatectomy. The preoperative De Ritis ratio can be applied to stratify the prognosis of patients with defined clinical conditions, thus allowing for an optimal individualized treatment plan.

## Data Availability Statement

The datasets generated or analyzed during this study are available from the corresponding authors on reasonable request. Requests to access these datasets should be directed to HY, yuhongping@stu.gxmu.edu.cn.

## Ethics Statement

The studies involving human participants were reviewed and approved by the Ethics Committee of the Guangxi Medical University Cancer Hospital. The patients/participants provided their written informed consent to participate in this study.

## Author Contributions

HY and WT contributed to the conception and supervision of this work. QM analyzed the data and wrote the manuscript. YL, ZZ, and RL reviewed and edited the manuscript. WG and BX did the validation of the analysis. All authors have contributed to the creation of this manuscript and approved the final manuscript.

## Funding

Funding was obtained through the following grants: National Natural Science Foundation of China (81660567), Key Research and Development Project of Guangxi (AA18221001, AB18050020), Key Project of Guangxi Natural Science Foundation (2018GXNSFDA050012), Shanghai Wu MengChao Medical Science Foundation (JJHXM-2019042), and Key Laboratory of Early Prevention and Treatment for Regional High Frequency Tumor, Ministry of Education (GKE-ZZ202104).

## Conflict of Interest

The authors declare that the research was conducted in the absence of any commercial or financial relationships that could be construed as a potential conflict of interest.

## Publisher’s Note

All claims expressed in this article are solely those of the authors and do not necessarily represent those of their affiliated organizations, or those of the publisher, the editors and the reviewers. Any product that may be evaluated in this article, or claim that may be made by its manufacturer, is not guaranteed or endorsed by the publisher.
