# Supplementary material for: Prognostic Value of Aspartate Transaminase/Alanine Transaminase Ratio in Patients With Hepatitis B Virus-Related Hepatocellular Carcinoma Undergoing Hepatectomy
Source: Front Oncol. 2022 May 18;12:876900. doi: 10.3389/fonc.2022.876900 (PMC9157420; doi:10.3389/fonc.2022.876900)
Supplement: Supplementary file 1 [file Table_1.docx]

Supplementary Materials

Table S1. Baseline characteristics of the study patients in the training cohort and validation cohort.

| Characteristics | Training cohort  (N = 803) | Validation cohort  (N = 344) | *P* value |
| --- | --- | --- | --- |
| Age [years, Mean (SD)] | 49.6 (10.9) | 49.8 (10.9) | 0.354 |
| Male, n (%) | 704 (87.7) | 307 (89.2) | 0.450 |
| Ethnicity, n (%) |  |  | 0.045 |
| Han | 477 (59.4) | 226 (65.7) |  |
| Others | 326 (40.6) | 118 (34.3) |  |
| Smoking, n (%) | 319 (39.7) | 124 (36.1) | 0.241 |
| Alcohol consumption, n (%) | 268 (33.4) | 99(28.8) | 0.126 |
| DM, n (%) | 35 (4.4) | 15 (4.4) | 0.999 |
| Liver cirrhosis, n (%) | 585 (72.8) | 246 (71.5) | 0.642 |
| BMI [kg/m^2^, Mean (SD)] | 22.4 (3.3) | 22.3 (3.1) | 0.651 |
| Leukocyte [10^9^/L, Mean (SD)] | 6.4 (2.1) | 6.4 (2.1) | 0.862 |
| Hemoglobin [g/L, Mean (SD)] | 130.4 (56.6) | 133.1 (33.9) | 0.399 |
| Albumin [g/L, Mean (SD)] | 39.3 (4.9) | 39.8 (5.5) | 0.156 |
| TBIL [μmol/L, Mean (IQR)] | 13.4 (9.9 - 17.8) | 13.0 (9.6 - 17.3) | 0.499 |
| ALBI |  |  | 0.852 |
| grade 1 | 413 (51.4) | 183 (53.2) |  |
| grade 2 | 382 (47.6) | 158 (45.9) |  |
| grade 3 | 8 (1.0) | 3 (0.9) |  |
| AST [U/L, Mean (IQR)] | 41.0 (32.0 - 58.0) | 39.0 (29.0 - 58.5) | 0.047 |
| ALT [U/L, Mean (IQR)] | 37.0 (26.0 - 55.0) | 35.5 (26.0 - 53.5) | 0.690 |
| HBV DNA [copies/mL, n (%)] |  |  | 0.232 |
| <10^3^ | 258 (32.1) | 123 (35.8) |  |
| $\text{≥}$10^3^ | 545 (67.9) | 221 (64.2) |  |
| Child–Pugh, n (%) |  |  | 0.979 |
| A | 756 (94.2) | 324 (95.7) |  |
| B | 47 (5.9) | 20 (5.8) |  |
| AFP [ng/ml, n (%)] |  |  | 0.179 |
| <400 | 464 (57.8) | 184 (53.5) |  |
| $\text{≥}$400 | 339 (42.2) | 160 (46.5) |  |
| Table S1. Continued. |  |  |  |
| PT [s, n (%)] |  |  | 0.495 |
| ≤14 | 667 (83.1) | 280 (81.4) |  |
| >14 | 136 (16.9) | 64 (16.6) |  |
| Tumor number, n (%) |  |  | 0.836 |
| Solitary | 607 (75.6) | 262 (76.2) |  |
| Multiple | 196 (24.4) | 91 (23.8) |  |
| Main tumor size [cm, n (%)] |  |  | 0.993 |
| <5 | 285 (35.5) | 122 (35.5) |  |
| $\text{≥}$5 | 518 (64.5) | 222 (64.5) |  |
| BCLC stage, n (%) |  |  | 0.350 |
| 0-A | 389 (48.4) | 177 (51.5) |  |
| B-C | 414 (51.6) | 167 (48.6) |  |
| Tumor capsule, n (%) | 539 (67.1) | 233 (67.7) | 0.840 |
| Tumor thrombus, n (%) | 229 (28.5) | 91 (26.5) | 0.475 |
| De-Ritis ratio | 1.1 (0.85 - 1.56) | 1.1 (0.86 - 1.42) | 0.116 |

Abbreviations: OS, overall survival; IQR, interquartile range; SD, standard deviation; DM, diabetes mellitus; AFP, alpha-fetoprotein; PT, prothrombin time; BMI, body mass index; TBIL, total bilirubin; ALBI, albumin-bilirubin; AST, aspartate transaminase; ALT, alanine transaminase; HBV, hepatitis B virus; BCLC, Barcelona Clinic Liver Cancer; De-Ritis ratio, aspartate transaminase/alanine transaminase ratio.
